# Supplementary figures and images for: Genome-Wide CRISPR-Cas9 Screen Reveals the Importance of the Heparan Sulfate Pathway and the Conserved Oligomeric Golgi Complex for Synthetic Double-Stranded RNA Uptake and Sindbis Virus Infection
Source: mSphere. 2020 Nov 11;5(6):e00914-20. doi: 10.1128/mSphere.00914-20 (PMC7657590; doi:10.1128/mSphere.00914-20)

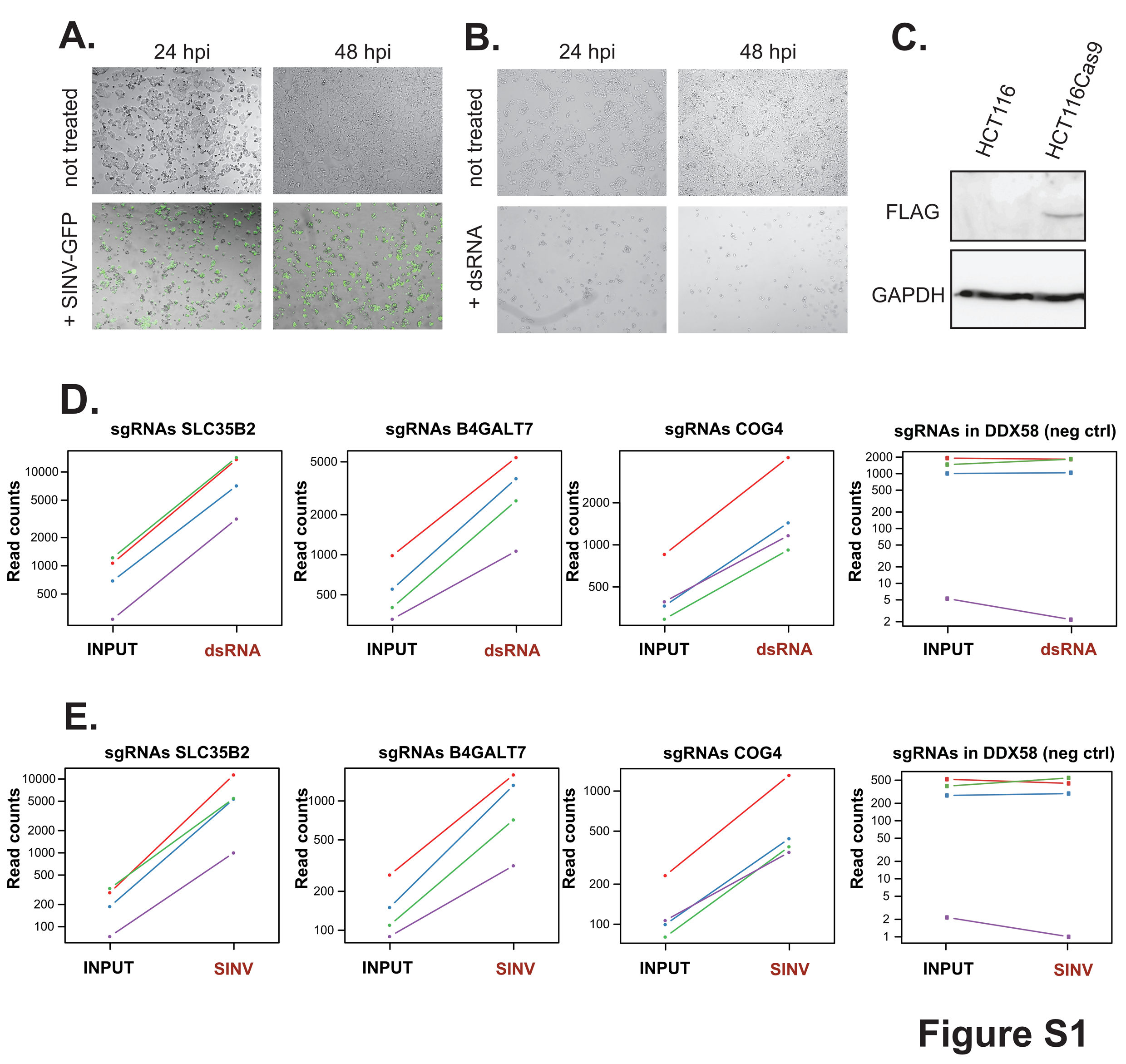

Supplement: FIG S1 [file mSphere.00914-20-sf001.tif]

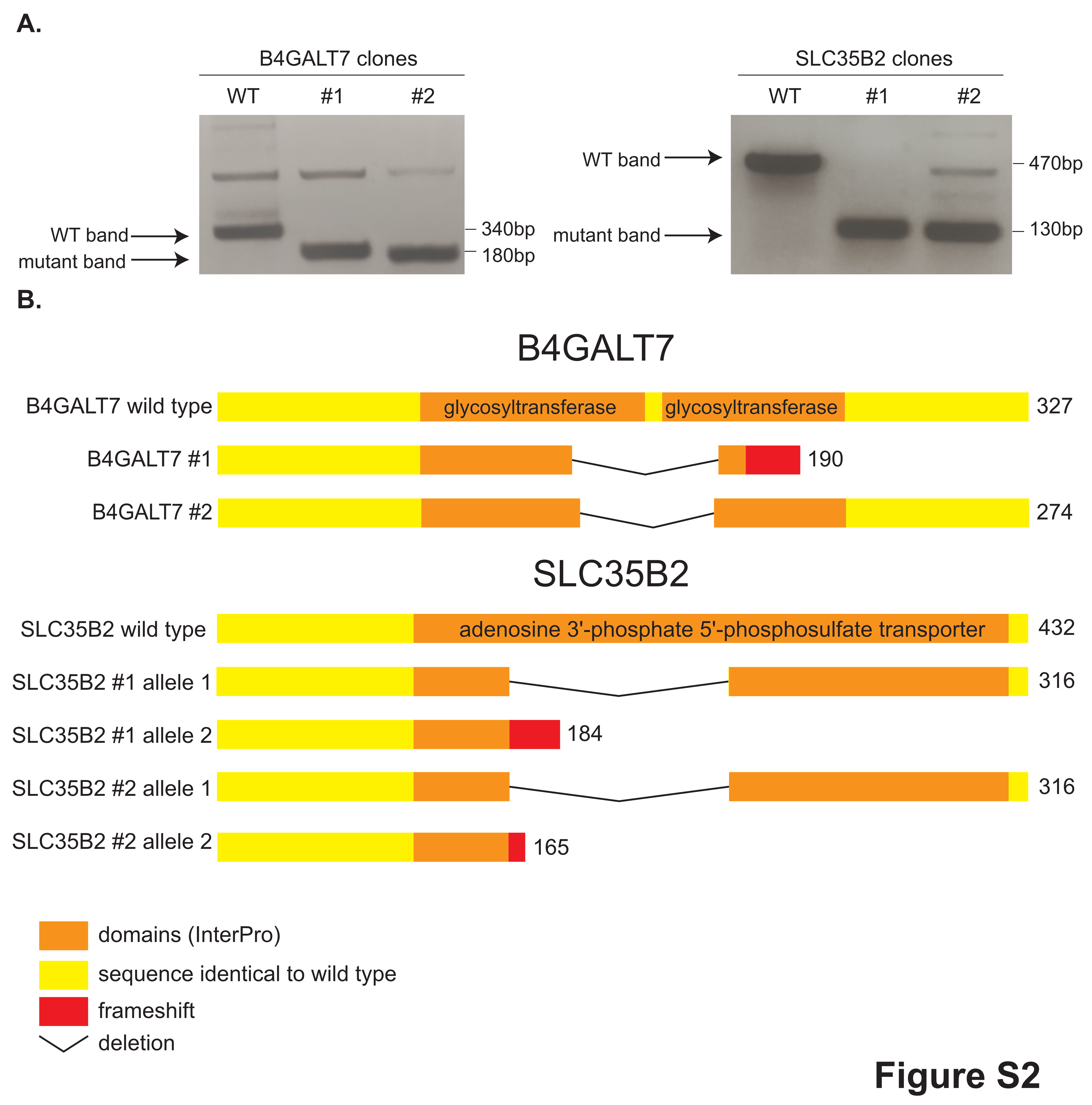

Supplement: FIG S2 [file mSphere.00914-20-sf002.tif]

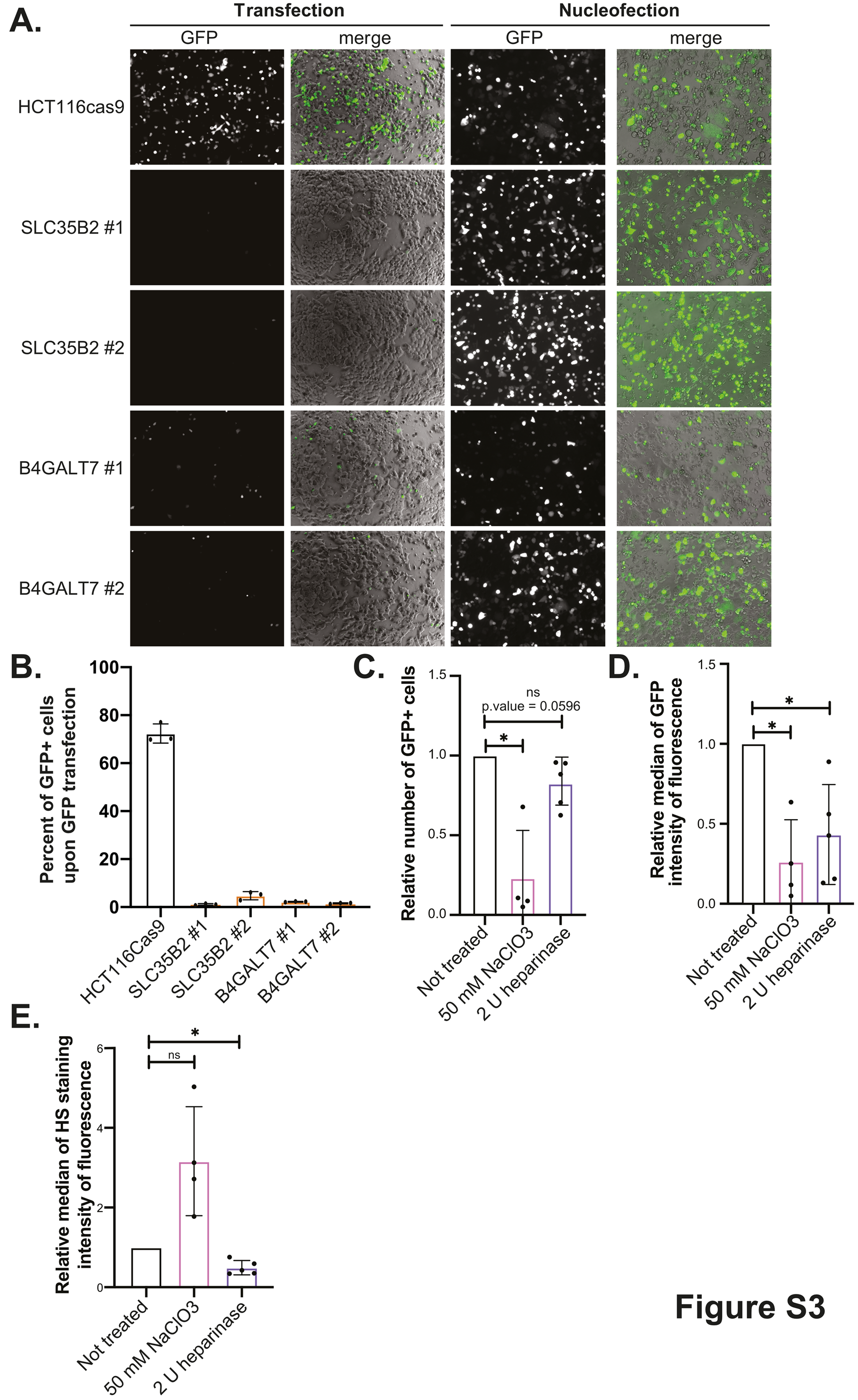

Supplement: FIG S3 [file mSphere.00914-20-sf003.tif]

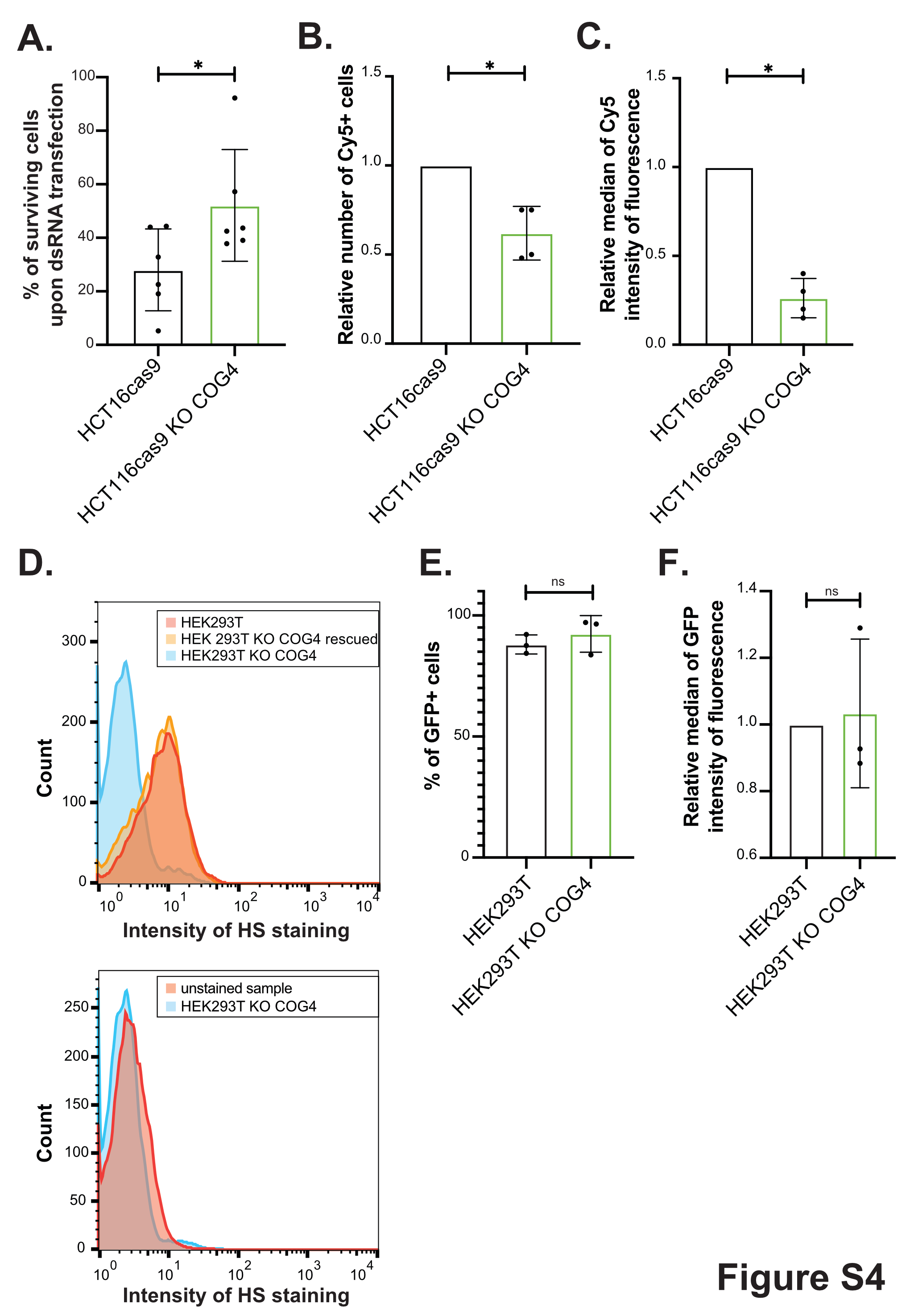

Supplement: FIG S4 [file mSphere.00914-20-sf004.tif]

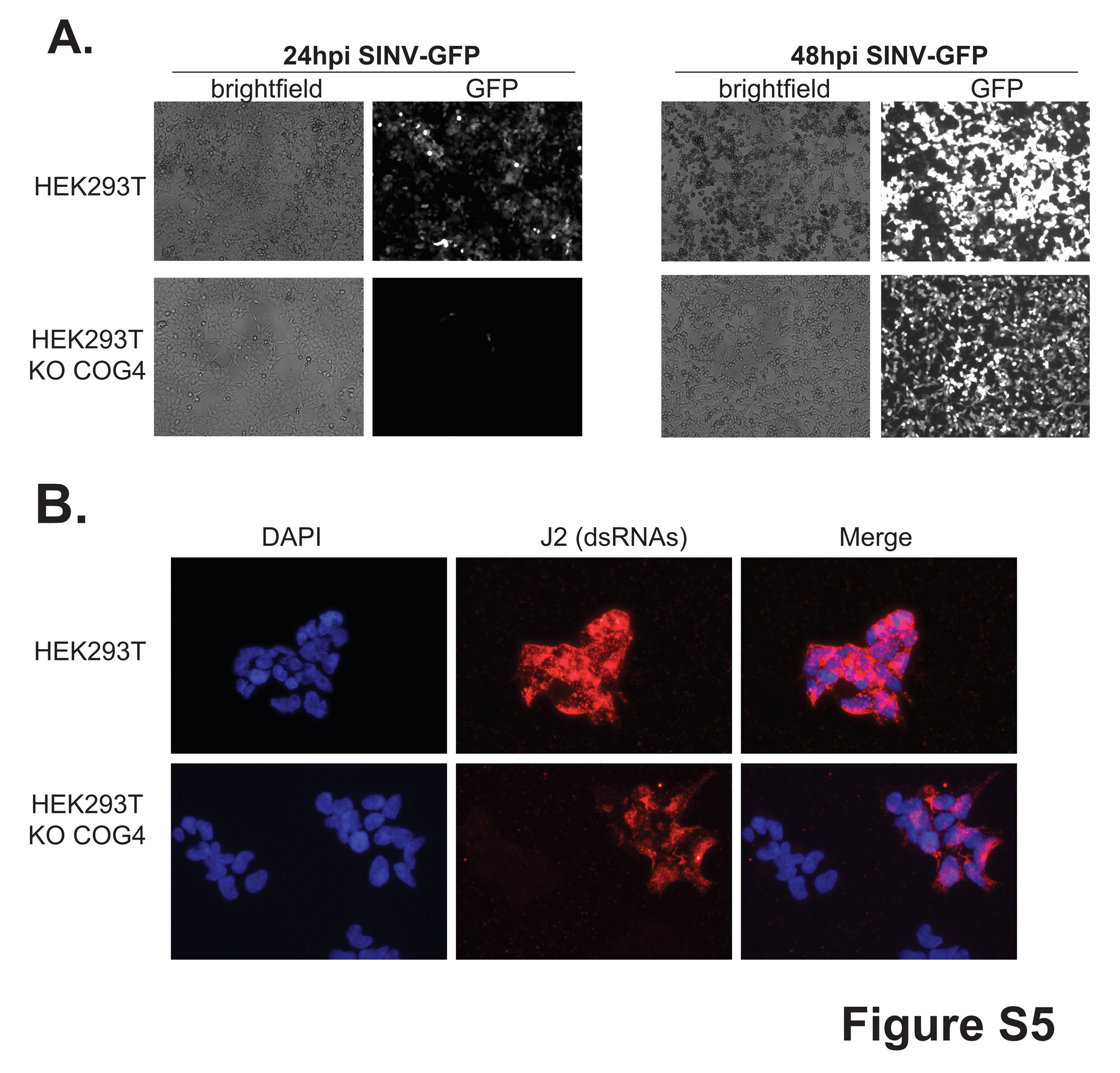

Supplement: FIG S5 [file mSphere.00914-20-sf005.tif]
