# Supplementary material for: Genome-Wide CRISPR-Cas9 Screen Reveals the Importance of the Heparan Sulfate Pathway and the Conserved Oligomeric Golgi Complex for Synthetic Double-Stranded RNA Uptake and Sindbis Virus Infection
Source: mSphere. 2020 Nov 11;5(6):e00914-20. doi: 10.1128/mSphere.00914-20 (PMC7657590; doi:10.1128/mSphere.00914-20)
Supplement: TABLE S1 [file mSphere.00914-20-st001.pdf]

Type	Primer name	Sequence (5' -> 3')
gRNA sequences	B4GALT7 gRNA sequence 1	CACTACAAGACCTATGTCGG
	B4GALT7 gRNA sequence 2	CGGGCAGCGCTCATCAACGT
	SLC35B2 gRNA sequence 1	GCACTCGGTTCATTAGCACC
	SLC35B2 gRNA sequence 2	TATAACCTGCCAGTAAGATG
	COG4 gRNA sequence 1	CAAAGTTCGTCAGCTTGACC
	COG4 gRNA sequence 2	ATGGTCACTCTCCACCGAAT
PCR primer to screen KO	B4GALT7 KO forward	AGTCAGTGCTGGGCCAGAGG
	B4GALT7 KO reverse	CAGCCGGTAGTGCTGCTTGG
	SLC35B2 KO forward	GGGGCCACAGCCACATCACC
	SLC35B2 KO reverse	AGGCAAACAGGGCATCCTGC
CRISPR screen primers	Crispr_lib_Seq_F01	AATGATACGGCGACCACCGAGATCTACACTCTTTCCCTACACGACGCTCTTCCG ATCTTTGTGGAAAGGACGAAACACCG
	Crispr_lib_Seq_F02	AATGATACGGCGACCACCGAGATCTACACTCTTTCCCTACACGACGCTCTTCCG ATCTCTTGTGGAAAGGACGAAACACCG
	Crispr_lib_Seq_F03	AATGATACGGCGACCACCGAGATCTACACTCTTTCCCTACACGACGCTCTTCCG ATCTGCTTGTGGAAAGGACGAAACACCG
	Crispr_lib_Seq_F04	AATGATACGGCGACCACCGAGATCTACACTCTTTCCCTACACGACGCTCTTCCG ATCTAGCTTGTGGAAAGGACGAAACACCG
	Crispr_lib_Seq_F05	AATGATACGGCGACCACCGAGATCTACACTCTTTCCCTACACGACGCTCTTCCG ATCTCAACTTGTGGAAAGGACGAAACACCG
	Crispr_lib_Seq_F06	AATGATACGGCGACCACCGAGATCTACACTCTTTCCCTACACGACGCTCTTCCG ATCTTGCACCTTGTGGAAAGGACGAAACACCG
	Crispr_lib_Seq_F07	AATGATACGGCGACCACCGAGATCTACACTCTTTCCCTACACGACGCTCTTCCG ATCTACGCAACTTGTGGAAAGGACGAAACACCG
	Crispr_lib_Seq_F08	AATGATACGGCGACCACCGAGATCTACACTCTTTCCCTACACGACGCTCTTCCG ATCTGAAGACCCTTGTGGAAAGGACGAAACACCG
	Crispr_lib_R01	CAAGCAGAAGACGGCATACGAGATAAGTAGAGGTGACTGGAGTTCAGACGTGT GCTCTTCCGATCTTCTACTATTCTTTCCCCTGCACTGT
	Crispr_lib_R02	CAAGCAGAAGACGGCATACGAGATACACGATCGTGACTGGAGTTCAGACGTGT GCTCTTCCGATCTTCTACTATTCTTTCCCCTGCACTGT
	Crispr_lib_R03	CAAGCAGAAGACGGCATACGAGATCGCGCGGTGTGACTGGAGTTCAGACGTGT GCTCTTCCGATCTTCTACTATTCTTTCCCCTGCACTGT
	Crispr_lib_R04	CAAGCAGAAGACGGCATACGAGATCATGATCGGTGACTGGAGTTCAGACGTGT GCTCTTCCGATCTTCTACTATTCTTTCCCCTGCACTGT
	Crispr_lib_R05	CAAGCAGAAGACGGCATACGAGATCGTTACCAGTGACTGGAGTTCAGACGTGT GCTCTTCCGATCTTCTACTATTCTTTCCCCTGCACTGT
	Crispr_lib_R06	CAAGCAGAAGACGGCATACGAGATTCCTTGGTGTGACTGGAGTTCAGACGTGT GCTCTTCCGATCTTCTACTATTCTTTCCCCTGCACTGT
	Crispr_lib_R07	CAAGCAGAAGACGGCATACGAGATAACGCATTGTGACTGGAGTTCAGACGTGT GCTCTTCCGATCTTCTACTATTCTTTCCCCTGCACTGT
	Crispr_lib_R08	CAAGCAGAAGACGGCATACGAGATACAGGTATGTGACTGGAGTTCAGACGTGT GCTCTTCCGATCTTCTACTATTCTTTCCCCTGCACTGT
	Crispr_lib_R09	CAAGCAGAAGACGGCATACGAGATAGGTAAGGGTGACTGGAGTTCAGACGTGT GCTCTTCCGATCTTCTACTATTCTTTCCCCTGCACTGT
dsRNA production	dsRNA Positive strand	GAAATTAATACGACTCACTATAGGCCATGCCCGAAGGCTACGTC
		TGTCGGCCATGATATAGACG
	dsRNA Negative strand	CCATGCCCGAAGGCTACGTC
		GAAATTAATACGACTCACTATAGGTGTCGGCCATGATATAGACG
qPCR primers	qPCR GAPDH	5’CCAGTGAGCTTCCCGTTCAG’3
		5’CTTTGGTATCGTGGAAGGACT’3
	qPCR dsRNA	GAACCGCATCGAGCTGAA
		CTACAACAGCCACAACGTCTA
	qPCR IFN-B	AAGGCCAAGGAGTACAGTC
		ATCTTCAGTTTCGGAGGTAA
